# Revisiting the Frequency and Antimicrobial Resistance Patterns of Bacteria Implicated in Community Urinary Tract Infections

**DOI:** 10.3390/antibiotics11060768

**Published:** 2022-06-03

**Authors:** Andreia Silva, Elisabeth Costa, Américo Freitas, Adelaide Almeida

**Affiliations:** 1 Department of Biology & CESAM, University of Aveiro, 3810-193 Aveiro, Portugal; andreiacms@ua.pt; 2 Clinical Analysis Laboratory Avelab, Rua Cerâmica do Vouga, 3800-011 Aveiro, Portugal; elisabeth.costa@avelab.pt (E.C.); americofreitas@netcabo.pt (A.F.)

**Keywords:** urinary tract infection, community acquired infections, uropathogens, antimicrobials, antimicrobial resistance

## Abstract

Urinary tract infections (UTIs) are one of the most common infectious diseases at the community level. The continue misuse of antimicrobials is leading to an increase in bacterial resistance, which is a worldwide problem. The objective of this work was to study the incidence and pattern of antimicrobial resistance of the main bacteria responsible for UTI in the community of central and northern Portugal, and establish an appropriate empirical treatment. The urine samples were collected in Avelab—Laboratório Médico de Análises Clínicas over a period of 5 years (2015–2019). The urine cultures were classified as positive when bacterial growth was equal to or higher than 10^5^ CFU/mL, and only for these cases, an antimicrobial susceptibility test was performed. Of the 106,019 samples analyzed, 15,439 had a urinary infection. Urinary infections were more frequent in females (79.6%) than in males (20.4%), affecting more elderly patients (56.9%). *Escherichia coli* (70.1%) was the most frequent uropathogen, followed by *Klebsiella pneumoniae* (8.9%). The bacteria responsible for UTI varied according to the patient’s sex, with the greatest differences being observed for *Enterococcus faecalis* and *Pseudomonas aeruginosa*, these being more prevalent in men. In general, there was a growth in bacterial resistance as the age of the patients increased. The resistance of bacteria in male patients was, in most cases, statistically different (Chi-Square test, *p* < 0.05) from that observed for bacteria isolated from female patients, showing, in general, higher resistance in male patients. Although *E. coli* was the most responsible uropathogen for UTI, it was among the bacteria most susceptible to antibiotics. The isolates of *K. pneumoniae*, *Proteus vulgaris* and *Enterobacter* showed high resistance to the tested antimicrobials. The most common multidrug-resistant (MDR) bacteria implicated in UTI were *K. pneumoniae* (40.4%) and *P. aeruginosa* (34.7%), but *E. coli*, the most responsible bacteria for UTI, showed a MDR of 23.3%. When we compared our results with the results from 10 years ago for the same region, in general, an increase in bacterial resistance was observed. The results of this study confirmed that urinary tract infections are a very common illness, caused frequently by resistant uropathogens, for which the antibiotic resistance profile has varied over a short time, even within a specific region. This indicates that periodically monitoring the microbial resistance of each region is essential in order to select the best empirical antibiotic therapy against these infections, and prevent or decrease the resistance among uropathogenic strains.

## 1. Introduction

Urinary tract infections (UTIs) affect approximately 150 million people worldwide every year, and are among the most frequent bacterial diseases [1]. With the high number of existing urinary infections, the high economic impact of their diagnosis and treatment is to be expected, resulting in great costs for health care annually [2]. UTI are considerably more common in women than in men, due to anatomic and physiological reasons. The vaginal cavity and rectal opening (where potential uropathogens live) are closer to the urethral opening in females, plus, on entering the urethra, bacteria are more likely to rise to the female bladder than the male bladder, due to the smaller urethral length [3,4]. These infections can be distinguished into lower UTIs (cystitis) and upper UTIs (pyelonephritis), with the clinical situation of the patient being more serious the microorganism’s invasion of the urinary tract becomes higher [5]. UTIs can also be clinically classified as complicated and uncomplicated. Uncomplicated UTIs are usually found in patients with a healthy urinary tract system, which is frequently seen in community-acquired infections, while patients with a defective or obstructive urinary tract system or patients who use medical devices such as catheters are clinically classified as having a complicated UTI, for which treatment is known to be more challenging [6]. The most common cause of UTI are the Gram-negative *Escherichia coli*, causing approximately 70–80% of community-acquired infections [6,7,8]. Besides *E. coli*, the most frequent bacteria found in UTIs are *Klebsiella pneumonia*, *Proteus mirabilis*, *Enterococcus faecalis*, *Pseudomonas aeruginosa*, *Staphylococcus saprophyticus*, *Staphylococcus aureus* and *Streptococcus agalactiae* [2,3,9,10].

The basic therapy of UTI is centred on the use of antibiotics. Antibiotics should be properly selected, taking into account factors such as the infectious bacteria’s antimicrobial susceptibility pattern, the type of infection (community-acquired or hospital-acquired) and some patient conditions including age, sex, previous antibiotic consumption, history of previous UTIs and the location of the UTI [6]. However, generally, antibiotics are prescribed empirically, with the intention of starting therapy as fast as possible. The right antibiotic prescription is critical, since if not chosen correctly, the antibiotic may not only fail to destroy bacteria reservoirs but may also act as a shelter for the survival of bacteria in bladder cells [11]. Currently, the antibiotics for uncomplicated cystitis suggested by the European Association of Urology (EAU) are fosfomycin, nitrofurantoin and pivmecillinam as the drugs of first choice when available [12]. Recommended alternative antimicrobials include trimethoprim-sulfamethoxazole (SXT) and cephalosporins if local resistance is lower than 20% [12]. Aminopenicillins in combination with a beta-lactamase inhibitor, such as amoxicillin plus clavulanic acid, are not so effective as short-term therapy but can still be used in select cases for alternative therapy [12]. For pyelonephritis, the most used antimicrobial agents for empirical oral treatment are the quinolones and cephalosporins, since they can reach adequate renal tissue levels [12].

Even though antibiotic resistance has been increasing over the past years all over the world, resistance patterns are variable, depending on the patient population and geographic region [3,8]. One of the factors to be considered when antibiotic therapy is started empirically is the regional prevalence of antimicrobial multidrug-resistant (MDR) bacteria (bacteria resistant to three or more antimicrobial classes [13]) among the common pathogenic bacteria. Therefore, monitoring this information periodically is very important because it reflects the changes over the years and it helps to decrease the number of failures during therapy [14]. The aim of this work was to investigate the incidence and pattern of antimicrobial resistance of the main bacteria responsible for UTI in the community of central and northern of Portugal within a five-year period (2015–2019).

## 2. Materials and Methods

### 2.1. Samples

All urine samples from patients in an ambulatory regime of the north and central region were analysed at Avelab Laboratório Médico de Análises Clínicas (Aveiro, Portugal), during the period of 2015–2019. The samples were collected from patients presenting clinical symptoms of UTI, pregnant women (including routine examinations throughout pregnancy, since women are more debilitated and are more likely to be infected), urinary tract infection post-treatment patients (patients who already used antibiotics empirically before collecting the sample) and asymptomatic patients who had been subjected to routine urine analysis during the study period, including multiple urine cultures of some patients. The following data were registered for each patient: sex, age, urine culture results, identification of the bacterial strain responsible for the UTI and the corresponding antimicrobial susceptibility test (AST) results.

Early urine was collected by the midstream clean-catch technique after the patient’s daily hygiene practices. The initial portion of the micturition was discarded and the midstream urine was collected directly into the sterile recipient. A collection bag surrounding the enthral area was used for collecting the urine from children under two years old. The bag was under control every 15 minutes, and after micturition, the bag was removed, sealed and stored at 4 °C until processing. The samples were analysed within one hour after collection; when this was not possible, the samples were stored at 4 °C until processing.

### 2.2. Microscopic Examination

Samples were homogenized and transferred to a conical tube of 10 mL. Secondly, the urine was centrifuged at 2500 rpm for five minutes and the supernatant was decanted. Lastly, the pellet was homogenized and placed onto slides that were directly examined to search for the presence of bacteria, leucocytes, erythrocytes, cells and crystals. Slides were also stained by the Gram technique to differentiate Gram-positive and Gram-negative bacteria.

### 2.3. Urine Culture

Different culture media were used and inoculated. A calibrated loop of 1 μL was dipped in a vertical position in the urine, and the loop was used to inoculate the media using the streak plate method. The Levine medium (Biokar Diagnostics, Allonne, France, BK056HA) was used for the detection of aerobic Gram-negative bacilli. For Gram-positive cocci, the urine samples were spread in mannitol salt agar (Biokar Diagnostics, BK030HA) for the detection of *Staphylococcus*, in blood agar (Bio-Rad, Hercules, CA, USA, 63784) for the detection of *Streptococcus* and in bile esculin agar (BD, Eysins, Switzerland, 212205) for the detection of *Enterococcus faecalis*.

The plates were incubated for 18–24 h at 37 °C. The plates of blood agar were incubated in a 5–10% CO_2_; atmosphere, while the others were incubated in a O_2_; atmosphere. After incubation, the samples were classified as positive, negative or contaminated. Contaminated classification was determined when polymorphic bacterial growth (growth of three or more bacterial species) was observed. When growth was lower than 10^5^ CFU/mL, the urine cultures were categorized as negative. The urine cultures were classified as positive when bacterial growth was equal to or higher than 10^5^ CFU/mL, and for these cases only, the antimicrobial susceptibility test was carried out.

### 2.4. Identification of Bacterial Isolates

For the identification of the bacterial isolates, further biochemical tests were carried out when the urine culture was positive. These identifications were based on the morphology of the isolated bacteria, the biochemical profile and the results of a microscopic examination of the Gram-stained smear. The following media were used to differentiate Enterobacteriaceae: Kigler (BD, 211317), tryptone (BD, 264410), Simmons citrate (BD, 211620) and urea (Oxoid, Madrid, Spain, CM00539). The catalase test was used to distinguish *Staphylococcus* from *Streptococcus*. The coagulase test (Biomérieux, Linda-a-Velha, Portugal, Slidex Staph plus, 73115) was used to identify *Staphylococcus aureus*. *Staphylococcus saprophyticus* was identified using the novobiocin susceptibility test (BD, 231314). The identification of Streptococcus agalactiea was achieved with the chromogenic medium Granada (Biomérieux). To identify Pseudomonaceae, the oxidase test (BD, 231746) was used. *Pseudomonas aeruginosa* was also identified by the production of diffusible pigments on Mueller–Hinton agar (Biokar Diagnostics, BK048HA) and a grape-like odour being released.

The reference strains *Escherichia coli* ATCC 25922, *Klebsiella pneumoniae* ATCC 13883, *S. aureus* ATCC 29123, *P. aeruginosa* ATCC 27853, *S. agalactiae* ATCC 13813, *E. faecalis* ATCC 29212 and *Proteus mirabilis* ATCC 35659 were used as positive controls.

### 2.5. Antimicrobial Susceptibility

The modified Kirby–Bauer disk diffusion method was used for the AST. A bacterial suspension was prepared in a physiological saline solution, with a turbidity of 0.5 on McFarland’s scale, using 1–2 colonies from pure cultures. For spreading the suspension on Mueller–Hinton agar, a swab was used. Antimicrobial-impregnated disks (BD BBL, Sensi-Disc) were placed onto the cultures’ Mueller–Hinton surface using an automated disk dispenser. For *E. coli*, the antibiotics amoxicillin, amoxicillin-clavulanic acid (AMX-CLA), cefazolin, cefuroxime, cefotaxime, nitrofurantoin, fosfomycin, ciprofloxacin, trimethoprim-sulfamethoxazole (SXT) and amikacin were tested. For other Enterobacteriaceae, the antibiotics tested were the same as those for *E. coli* except for fosfomycin. *P. aeruginosa* was tested for amikacin, gentamicin, tobramycin, ceftazidime, cefepime, aztreonam, piperacillin-tazobactam (PIP-TAZ) and ciprofloxacin. For *E. faecalis*, amoxicillin, ampicillin, gentamicin (high dose), nitrofurantoin, ciprofloxacin and levofloxacin were used. For S. agalactiae, cefotaxime, nitrofurantoin, ampicillin, amoxicillin, levofloxacin and SXT were tested. For Staphylococcus nitrofurantoin, amoxicillin-clavulanic acid, ciprofloxacin and SXT were used. For *S. aureus* and for *S. saprophyticus*, respectively, gentamicin and amoxicillin were also tested.

The AST plates were incubated at 37 °C for 18–24 h. Following incubation, the diameter of the zones of inhibition were measured to determine the antimicrobials’ efficacy [15]. According to the measured inhibition zone, the bacterial strains were classified as susceptible (S), intermediate (I) or resistance (R) [15], and uropathogens that were resistant to three or more antimicrobial classes were considered to be multidrug-resistant (MDR) [13].

### 2.6. Statistical Analysis

For the statistical analysis, the Statistical Package for the Social Sciences (SPSS) version 26.0 for Windows was used. To make the treatment of the data easier, the main bacteria responsible for UTI were selected, namely *E. coli*, *K. pneumoniae*, *P. mirabilis*, *E. faecalis*, *P. aeruginosa*, *S. aureus*, *S. agalactiae*, *Enterobacter* spp., *P. vulgaris*, *S. saprophyticus* and *Klebsiella oxytoca*. These selected bacteria represented 97.4% of all positive urines, while those not selected represented 2.6%. Consequently, from the 15,439 positive samples, 15,025 corresponded to samples contaminated with the selected bacteria (considered in the treatment data) and 414 samples corresponded to samples contaminated with the non-selected bacteria. Descriptive statistics, such as the mean and percentages, were calculated. For the categorical variables, a Chi-square test was used, and the significant level established was <0.05. The binomial test was used for categorial variables with two expressions (female and male), with the significance level established at <0.05.

## 3. Results

From the 5-year study period, 106,019 (from the 15,439 positive samples, excluding 414 samples corresponding to samples contaminated with the non-selected bacteria (2.6%)) samples of ambulatory patients were analysed, and 15,439 (14.6%) were positive for a urinary tract infection.

### 3.1. Characterization of Patients with Bacterial UTI

The female patients with UTI represented 79.0% in total, while males represented 21.0% of the samples. The age of the patients ranged from 1 to 106 years old, with a mean of 64.0 years. For female UTI patients, the average age was 62.3 years, while for male patients, it was 70.6 years. From the 15,025 positive bacteriological tests considered, 11,959 (79.6%) were from female patients and 3066 (20.4%) were from male patients (Table 1). The group most affected by UTI were the elderly, with a frequency of 56.9% (42.1% for females and 14.8% for males). The group least affected by UTI were adolescents, showing the lowest frequency, 1.0% (Table 1). Children were responsible for 1.9% of the infections, young adults for 8.5% and adults for 31.6% (Table 1). A higher prevalence of female infections was detected for all the age groups; however, males showed a higher prevalence in the elderly when compared with other age groups (Table 1).

### 3.2. Bacteria Implicated in UTI

**Figure 1:**
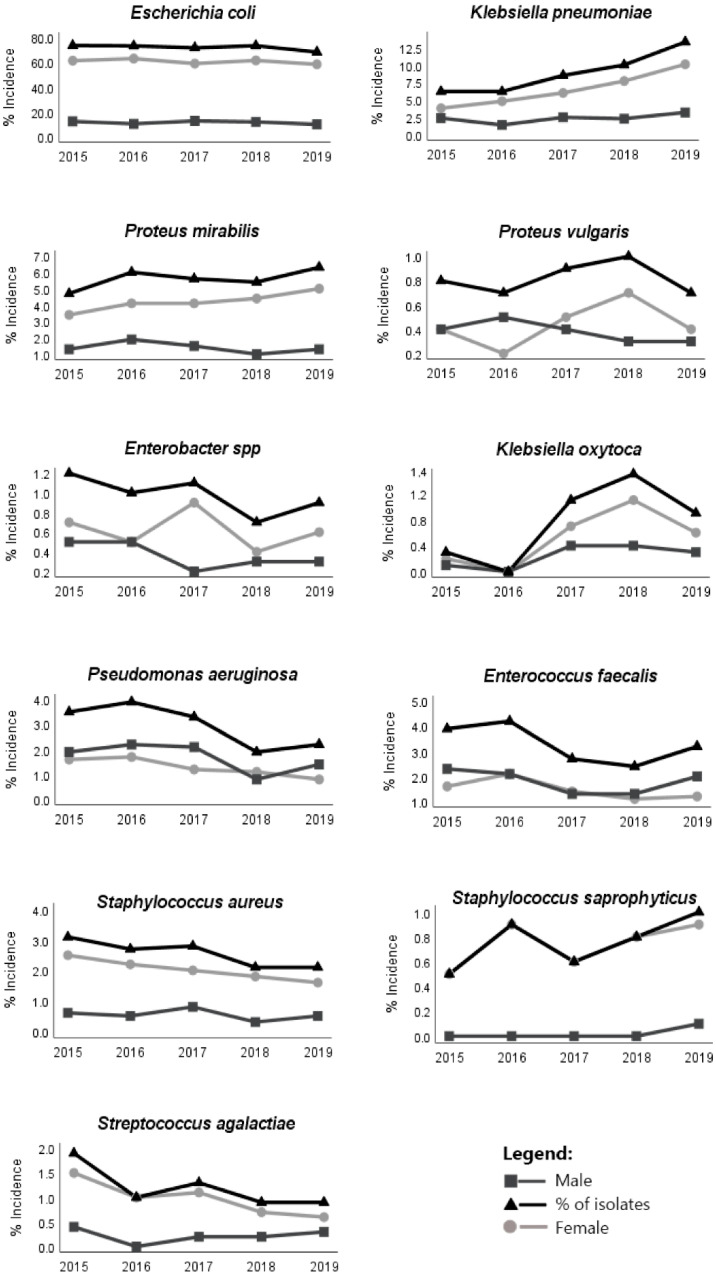
Incidence of the main bacteria implicated in UTIs by sex during the study period (2015–2019).

The 11 bacteria most implicated in the UTI during the 5-year study period corresponded to more than 95% of the bacterial isolates annually. The most predominant agents were *E. coli* (70.1%), *K. pneumoniae* (8.9%), *P. mirabilis* (5.5%), *E. faecalis* (3.2%), *P.* *aeruginosa* (2.8%), *S. aureus* (2.5%), *S. agalactiea* (1.1%), *Enterobacter* spp. (0.9%), *P. vulgaris* (0.8%), *S. saprophyticus* (0.8%) and *K. oxytoca* (0.8%) (Table 1). The incidence of the main bacteria responsible for UTI varied significantly (Chi-square test, *p* < 0.05) across the study period. Overall, the incidence of *K. pneumonia*, *K. oxytoca* and *S. saprophyticus* increased and the incidence of *S. agalactiea* and *P. aeruginosa* decreased (Figure 1). *E. coli* was always the pathogen most implicated in the UTI, followed by *K. pneumoniae* and *P. mirabilis*. The presence of the same bacteria between males and females was evident, but the relative proportions were different (binomial test *p* < 0.05). *E. coli* was the most present bacteria in both males and females, but females showed an average incidence of 76.2% and males had an average of 55.6% (Table 1). The incidence of *E. faecalis* was distinct between females and males, this bacterium being the third most responsible (8.8%) for UTI in males and only fifth (1.8%) in females (Table 1). The same occurred with *P. aeruginosa*, which was the fourth most common cause for males (8.1%) and the sixth for females (1.6%) (Table 1).

The incidence of bacteria in the different age groups increased significantly with the patient’s age (Chi-square test, *p* < 0.05). Significant differences (binomial test *p* < 0.05) were also observed when samples from females and males were analysed separately, showing that the difference also increased with the age (Table 1).

### 3.3. Antimicrobial Resistance Patterns of the Main Bacteria Implicated in UTI

In general, Gram-negative bacteria exhibited higher resistance than Gram-positive bacteria.

The Gram-negative bacteria, with the exception of *E. coli*, exhibited higher resistance to penicillins, quinolones, SXT, first- and second-generation cephalosporins, and nitrofurantoin when compared with the other tested antimicrobials (Table 2). With the exception of *P. aeruginosa*, which showed a resistance of 17.3% to amikacin, the studied bacteria showed the least resistance to amikacin and fosfomycin, corresponding to a resistance lower than 4.5% (Table 2). The isolates of *K. pneumoniae*, *P. vulgaris* and *Enterobacter* showed high resistance to several tested antimicrobials, and the isolates of *E. coli* showed the least resistance to the studied antibiotics (Table 2).

Among the Gram-positive bacteria, the highest resistance was observed for SXT, quinolones and amoxicillin (Table 3). *E. faecalis* showed high resistance to ciprofloxacin and levofloxacin (46.4% and 38.3%, respectively; Table 3). *S. agalactiea* presented low resistance to cefotaxime and to nitrofurantoin of less than 3 and 4%, respectively, but high resistance to SXT (Table 3).

During the study period, the bacterial resistance changed significantly (Chi-squared test, *p* < 0.05).

**Figure 2:**
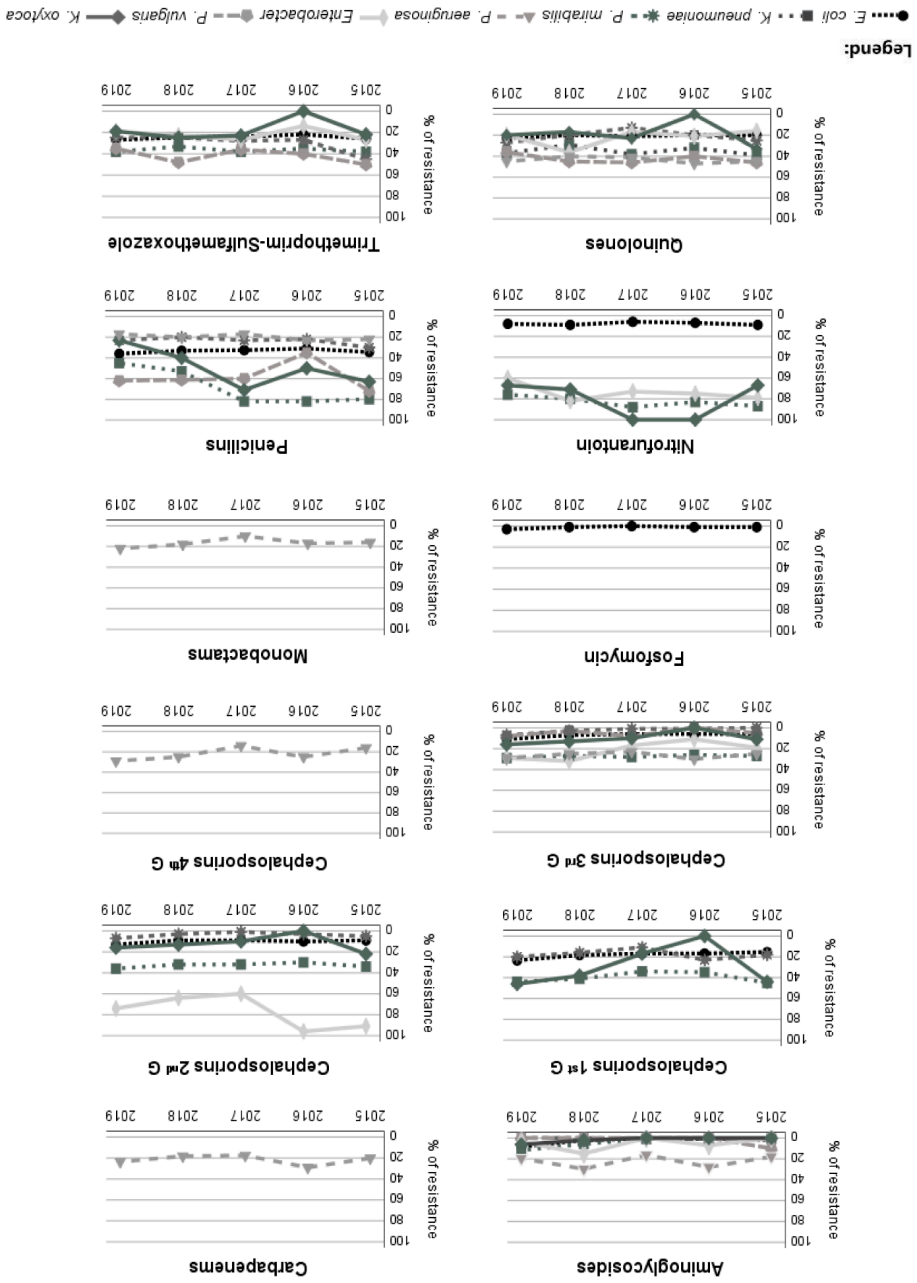
Variation in the antimicrobial resistance patterns of Gram-negative bacteria during the study period (2015–2019). G, generation.

**Figure 3:**
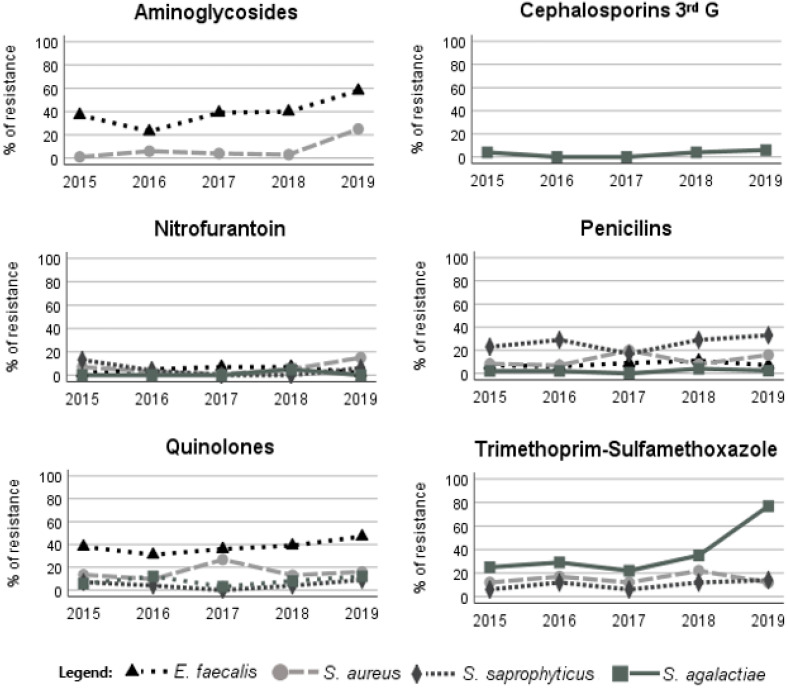
Variation in the antimicrobial resistance patterns of Gram-positive bacteria during the study period. G, generation.

The Gram-negative isolates of *E. coli* showed a slight increase in resistance to amikacin and cephalosporins of the first generation throughout the years (Figure 2). *Enterobacter* showed increased resistance to cephalosporins of the third generation, more precisely, to cefotaxime (Figure 2). *P. aeruginosa* showed a slight resistance increase to aztreonam. In general, the recorded resistance to ciprofloxacin was constant during the study period, but *K. oxytoca* isolates showed a slight decrease. *P. mirabilis* and *P. vulgaris* showed a decrease in SXT resistance (Figure 2). The Gram-positive bacterial isolates of *S. aureus* showed an increase in resistance to the aminoglycosides during the study period (Figure 3). *E. faecalis* and *S. aureus* also showed a slight increase in nitrofurantoin resistance, but *S. saprophyticus* presented a decrease in resistance to this antimicrobial (Figure 3). *E. faecalis* and *S. saprophyticus* showed an increase in resistance to penicillins. S. agalactiae isolates were the bacteria that presented the greatest increase in SXT resistance (Figure 3). The resistance of bacteria implicated in UTIs of male patients was, in most cases, statistically different (Chi-square test, *p* < 0.05) from that observed for bacteria isolated from female patients, showing, in general, higher resistance in male patients (Table 2 and Table 3).

### 3.4. Calculated Bacterial Resistance to Recommended Antimicrobials

Taking the values of drug resistance of each bacterium and its incidence into account, the resistance patterns were calculated according to the uropathogens’ incidence (multiplying the bacterium’s average resistance by its incidence) for the two first-line antibiotics indicated for treating UTI according to EAU (Table 4).

For *E. coli*, the resistance to the first-line therapy antimicrobials tested in this study was low: 1.4% and 7.0% for fosfomycin and nitrofurantoin, respectively. For the other uropathogenic bacteria, resistance was higher, in most cases (*E. faecalis*, *S. aureus*, S. agalactiae and *S. saprophyticus* being the exceptions), with an average resistance of 49.7% to nitrofurantoin. The calculated resistance for all bacteria was 1.0% for fosfomycin and 19.9% for nitrofurantoin (Table 4).

The resistance of *E. coli* to the alternative drugs was slightly higher for some antibiotics, being 20.5%, 20.3%, 24.8%, respectively, for the quinolones, AMX-CLA and SXT. Among the tested alternative drugs, *E. coli* showed the lowest resistance to cephalosporins: 14.9%, 10.0% and 7.2% for the first, second and third generations, respectively (Table 5). The other bacteria implicated in UTIs showed, on average, higher resistance to these drugs, having an average of 57.4%, 46.4%, 11.9%, 25.5%, 44.5% and 27.1% for first-, second- and third-generation cephalosporins, quinolones, AMX-CLA and SXT, respectively.

The resistance of all the 11 bacteria most implicated in UTI to the alternative drugs quinolones, AMX-CLA and SXT was 22.5%, 23.1% and 23.8%, respectively, being higher than the calculated resistance to the first-line antibiotics (Table 5). The calculated resistance to cephalosporins from the first, second and third generations (16.7%, 12.1%, 7.9%, respectively) was, however, smaller than that to nitrofurantoin (19.9%), a first-line antibiotic.

### 3.5. Multidrug-Resistant Bacteria Implicated in UTI

The percentage of multidrug-resistant (MDR) bacteria varied between 6% and 40%. The most common MDR bacteria implicated in UTI were *K. pneumoniae* (40.4%) and *P. aeruginosa* (34.7%) (Table 6). The most responsible bacteria for UTI, *E. coli*, showed a multidrug resistance of 23.3%. *S. saprophyticus* and *S. agalactiae* presented the least multidrug resistance (5.8% and 6.8%, respectively) (Table 6). No significant differences (Chi-square test, *p* < 0.05) in the incidence of MDR bacteria were observed during the study period. The incidence of MDR bacteria was higher in the elderly group (Chi-square test, *p* < 0.05).

## 4. Discussion

As already observed in other studies, *E. coli* was the most common bacteria implicated in UTI, being responsible for more than half of the infections (70.1%). *E coli* is part of the intestinal flora and therefore easily colonises the urinary tract, causing more frequently cystitis. *K. pneumoniae* (8.9%) and *P. mirabilis* (5.5%) were, respectively, the second and third uropathogens most implicated in UTI, as observed in other studies at the community level [16,17,18]. Even though *E. coli* was the most common uropathogen in both sexes, its incidence was significantly higher in women (76.2%) than in men (55.6%), likely due to anatomic and physiological reasons, since the length of the urethra is smaller for women, allowing the enterobacteria to rise to the bladder more easily. As observed in other studies [14,16,19], *P. aeruginosa* was one of the bacteria that contributed most to the differences between males and females, followed by *E. faecalis*. Both bacteria were more frequently associated with male infections (8.8% and 8.1% for *E. faecalis* and *P. aeruginosa*, respectively, versus 1.8% and 1.6% in females). Amna et al. [19] concluded that non-*E. coli* bacteria are more likely to infect men, attributing this to the fact that UTIs in men are frequently more complicated due to the use of catheters [20,21]. In fact, *Enterococcus* and *Pseudomonas* have been associated with infections related to catheters in the urinary tract [22,23,24,25].

As has been documented before [3,14,16,26], urinary tract infections increase with the patient’s age. The elderly group was responsible for more than half of the infections (56.9%), and a variety of reasons can explain this increase, since the elderly are more prone to frequent hospitalisations, which exposes them to nosocomial pathogens; residing in care facilities, the frequent use of antimicrobials, the frequent use of urogenital catheters, the decrease in adaptive and innate immunity, and previous cases of UTI [27].

If we compare our results to those of Linhares et al. [14], found in the same region ten years ago (between 2000 and 2009), the incidence of some of the main bacteria responsible for UTI changed. *E. coli* remained the principal uropathogen for the infections, but *S. aureus* decreased, passing from the second most responsible pathogen (incidence of 6.0%) to sixth (incidence of 2.5%) in the current study. The difference in the incidence of *S. aureus* may, however, be due to the detection method used, which changed between the two study periods. Another difference was in the incidence of *Klebsiella:* 10 years ago, this bacterium only represented 4.3% of the UTI, but in this study, *K. pneumonia* was the second most frequent bacteria (8.9%), which can be related to the increase in the resistance of this bacterium between the two study periods (e.g., 81.7% in this study versus 32.8% 10 years ago for nitrofurantoin and 34.6% in this study versus 22% 10 years ago for ciprofloxacin).

The incidence of *E. coli*, the most important uropathogen implicated in UTI, was constant during the period of the study. However, the incidence of *K. oxytoca* and *K. pneumonia* in UTI showed an increase over the course of the years. With the average life expectancy increasing, it is expectable that elderly patients’ hospitalization will increase as well, inducing a rise in the transmission of bacterial strains between hospital and the community. As *K. pneumonia* and *K. oxytoca* are commonly found in the hospital environment, this may explain the increase in both, since there was, in fact, an increase in these bacteria for the elderly group during the study period.

Gram-negative bacteria are, in general, more resistant than Gram-positive bacteria. The outer membrane of Gram-negative bacteria is the main reason for their resistance to a large range of antibiotics. Gram-negative outer membrane alterations, such as changes in the hydrophobic properties or mutations in the porins and other factors, can create resistance [28]. Gram-positive bacteria lack this important layer, making them less resistant to antibiotics than Gram-negative ones. The uropathogen responsible for more than half of the UTI, *E. coli*, was one of the bacteria that presented the lowest antimicrobial resistance. Its resistance to the first-line antimicrobials recommended by the EAU for uncomplicated cystitis UTI, fosfomycin (1.4%) and nitrofurantoin (7%), was considerably low; therefore, these drugs can be considered suitable for the empirical treatment of UTI in the studied community. However, *K. pneumonia*, the second most frequent bacteria, presented resistance to nitrofurantoin in 81.7% of the cases, a value higher than usual. Zanichelli et al. also observed the high resistance of *K. pneumonia* to nitrofurantoin in an 8-year study (2008–2016) [29]. Relative to the alternative antimicrobials recommended by the EAU, some of the studied antibiotics were adequate for treating UTI empirically in the community. The cephalosporins were effective against *E. coli*, as less than 20% of the isolates were resistant to this drug. Cefotaxime was the most effective against *E. coli*, with only 7.2% of the isolates being resistant, showing a resistance close to that of nitrofurantoin, a first-line antimicrobial. This antibiotic can be also a good option against other bacteria, since their resistance to this drug was low. If we consider the calculated resistance results, it can be suggested that cephalosporins may be a better option than nitrofurantoin (suggested as a first-line therapy) for empirical treatment, since its calculated resistance was low. According to Bonkat et al. [12], antibiotics can be used empirically against bacteria to treat cystitis when the resistance to these drugs is lower than 20%. In this study, the resistance of *E. coli* to SXT was 24.8%, being even higher in bacteria isolated from men (33.1%), so this antimicrobial should not be an option in this region. The resistance of *E. coli* to AMX-CLA was 20.3%, which is very close to the borderline. Similar bacterial resistance values close to 20% against these antibiotics has been observed in other studies [16,17,18].

The results of this study support the idea that the choice of empirical antimicrobial therapy should consider the sex of the patient. On average, uropathogens isolated from male patients registered a higher resistance to antimicrobials. Linhares et al. [14], in the study carried out in the same region tem years ago, also found significant differences in susceptibility patterns between women and men.

For the empirical treatment of pyelonephritis, the EAU recommends antimicrobials that can reach adequate renal tissue levels, such as quinolones and cephalosporins, if the resistance local rates are lower than 10%. This was not the case in this study for quinolones, as most of the uropathogens showed a resistance higher than 20%, indicating that this drug should not be prescribed empirically in cases of pyelonephritis. The cephalosporins can be an adequate option, but it should be taken into account that oral cephalosporins achieve significantly lower concentrations than intravenous cephalosporins [12].

The antimicrobial resistance, in general, as already observed in other studies, increased with age [14,16,18,30]. The main bacteria isolated in this study showed significant differences among the different age groups, probably because older patients are more likely to have recurring infections due to frequent hospitalizations, which allows the transmission of bacterial resistance between hospitals and the community. However, the increases in resistance vary by antibiotic class, possibly reflecting variations in the rates of the prescribed antibiotics [31]. Multidrug resistance is a risk factor for inappropriate empirical treatment, and it is associated with an increased mortality. This study detected high incidence of bacteria that were resistant to three or more antimicrobial classes. The bacteria most responsible for UTI, *E. coli* and *K. pneumoniae*, reached 23% and 40%, respectively, while *P. aeruginosa* showed 35% of MDR bacteria. A comparison of these results with a study carried out 10 years ago in the same region [14] indicated an increase in MDR bacteria. Linhares et al. [14] observed a rate of MDR of 17% for *E. coli*, 35% for *Klebsiella* spp. and 25% for *P. aeruginosa*. These results reinforce the problem of resistant bacteria in the community, a growing problem in our society. This increase in resistance is probably due to the misuse and/or overuse of antibiotics and transmission of resistance between the community and hospitals.

Even though *E. coli* did not show huge changes in antibiotic resistance when our results were compared with results from 10 years ago [14], we can see increases in resistance greater than 10% for cefazolin, cefuroxime, AMX-CLA, nitrofurantoin and ciprofloxacin for *K. pneumoniae* when compared with *Klebsiella* spp. Moreover, *P. aeruginosa* showed an increase of more than 10% in resistance to gentamicin, imipenem, ceftazidime, cefepime and aztreonam. *P. mirabilis* exhibited a rise in resistance greater than 10% for amoxicillin and SXT. *P. vulgaris* registered resistance rates at least 18% higher when compared with 10 years ago for AMX-CLA and ciprofloxacin, although the resistance of this bacterium to SXT decreased by 15% during the same period.

If we compare our results with those obtained ten years ago by Linhares et al. [14], this reveals an increase in age for the tested patients. The mean age of patients of this study was 64 years old, while for Linhares et al., it was 54 years old. In this study, more than half (56.9%) of the patients belonged to the elderly group, while for Linhares et al. [14], only 38.6% of the patients were from that age group. This may be one of the reasons for the increase in resistance, as older patients are associated with higher resistance to antimicrobials for the reasons described above, as the elderly are most likely to have frequent hospitalisations, transmitting bacterial resistance between the hospital and the community.

## 5. Conclusions

The results obtained in this study indicated that *E. coli* was the most prevalent uropathogen, being responsible for more than half of the urinary tract infections. As age increased, differences between females and males increased as well. Even though *E. coli* was the most prevalent uropathogen, this bacterium was one of the most susceptible to antibiotics. *E. coli* was susceptible to nitrofurantoin and fosfomycin, the first-line drugs indicated for treating uncomplicated UTI according to the EAU, but the same was not observed for nitrofurantoin in other Gram-negative uropathogens. Since *E. coli* is by far the uropathogen most responsible for cases of UTIs, these antibiotics can still be considered good choices for empirical therapy. However, when the clinical history of the patient indicates recent hospitalisations or previous cases of UTI by other Gram-negative isolates, nitrofurantoin should not be the first option. *E. coli* presented higher resistance to the alternative antibiotics SXT and AMX-CLA when compared with first-line antibiotics, but cephalosporins, such as cephalosporins from the second and third generation, can be a good alternative treatment. According to the EAU, SXT and AMX-CLA are not suitable for treating UTI empirically inpatients of the studied region.

The results of this study confirm that urinary tract infection is a very common illness caused frequently by resistant uropathogens, for which the antibiotic resistance profile varies over a short time, even within a specific region. This indicates that periodically monitoring the microbial resistance of each region is essential in order to select the best empirical antibiotic therapy against these infections and prevent or decrease the resistance among uropathogenic strains.

## Figures and Tables

**Table 1:** Incidence of the main bacteria implicated in urinary tract infections during the study period according to age and sex.

	Children			Adolescents			Young Adults			Adults			Elderly			Isolates in the 5 Years (%) ^1^ (N = 15,439)	Females (%) ^1^ (N = 11,959)	Males (%) ^1^ (N = 3066)
	0–12 Years			13–18 Years			19–34 Years			35–64 Years			>65 Years					
	Total ^1^	F ^2^	M ^2^	Total ^1^	F ^2^	M ^2^	Total ^1^	F ^2^	M ^2^	Total ^1^	F ^2^	M ^2^	Total ^1^	F ^2^	M ^2^			
Bacteria	N = 282	n = 232	n = 50	N = 157	n = 147	n = 10	N = 1277	n = 1206	n = 70	N = 4753	n = 4044	n = 709	N = 8556	n = 6330	n = 2226			
*E. coli*	74.8	67.4 ^3^	7.4	63.1	59.3 ^3^	3.8	75.3	71.1 ^3^	4.2	77.1 ^4^	67.2 ^3^	9.9	68.7	55.2 ^3^	13.5	70.1	76.2 ^3^	55.6
*K.* *pneumoniae*	1.8	1.1	0.7	6.4	5.8 ^3^	0.6	4.5	4.1 ^3^	0.4	6.2	4.9 ^3^	1.3	11.7 ^4^	8.3 ^3^	3.4	8.9	8.4	11.7 ^3^
*P. mirabilis*	16.3 ^4^	8.9	7.4	10.2	9.6 ^3^	0.6	5.3	5.2 ^3^	0.1	5.2	4.4 ^3^	0.8	5.5	3.0 ^3^	2.5	5.5	5.4	6.7 ^3^
*P. vulgaris*	1.1	0.4	0.7	0.6	0.6	0.0	0.1	0.1	0.0	0.4	0.2	0.2	1.2 ^4^	0.7	0.5	0.8	0.6	1.9
*Enterobacter* spp.	0.0	0.0	0.0	1.3 ^4^	1.3	0.0	0.4	0.4	0.0	0.8	0.6 ^3^	0.2	1.2	0.6	0.6	0.9	0.8	1.8 ^3^
*K. oxytoca*	0.4	0.0	0.4	0.0	0.0	0.0	0.3	0.2	0.1	0.5	0.4 ^3^	0.1	1.1 ^4^	0.7 ^3^	0.4	0.8	0.7	1.3 ^3^
*P.* *aeruginosa*	2.1	1.8	0.4	0.0	0.0	0.0	0.2	0.1	0.1	1.3	0.3 ^3^	1.3	4.2 ^4^	1.9	2.3	2.8	1.6	8.1 ^3^
*E. faecalis*	1.1	0.7	0.4	0.6	0.6	0.0	2.5	2.1 ^3^	0.4	2.1	1.2	0.9	4.1 ^4^	1.5 ^3^	2.6	3.2	1.8	8.8 ^3^
*S. aureus*	1.8	1.8	0.0	14.0 ^4^	12.7 ^3^	1.3	7.0	6.8 ^3^	0.2	3.5	3.2 ^3^	0.3	1.2	0.4 ^3^	0.8	2.5	2.5	2.8 ^3^
*S.* *saprophyticus*	0.7	0.7	0.0	3.2 ^4^	3.2	0.0	3.1	3.0 ^3^	0.1	1.3	1.3 ^3^	0.0	0.1	0.1	0.0	0.8	1.0 ^3^	0.2
*S. agalactiae*	0.0	0.0	0.0	0.6	0.6	0.0	1.3	1.2 ^3^	0.1	1.6 ^4^	1.4 ^3^	0.2	1.0	0.7 ^3^	0.3	1.1	1.2 ^3^	1.1
Total (%)	1.9	1.6	0.3	1.0	0.9	0.1	8.5	8.0	0.5	31.6	26.9	4.7	56.9	42.1	14.8	-	79.6	20.4

N: total number of bacteria for each age group; n: total number of bacteria for each sex; ^1^, percentage determined in relation to N; ^2^, percentage determined in relation to n; ^3^, statistically significant differences of frequency between sexes; ^4^ , statistically significant differences between age groups; M: male; F: female.

**Table 2:** Average antimicrobial resistance of the main Gram-negative uropathogens for female and male patients.

Antimicrobial Group		Antimicrobials	*E. coli*				*K. pneumoniae*				*P. mirabilis*				*P. vulgaris*				*Enterobacter* spp.				*K. oxytoca*				*P. aeruginosa*			
			N	%	F	M	N	%	F	M	N	%	F	M	N	%	F	M	N	%	F	M	N	%	F	M	N	%	F	M
Aminoglycosides		Amikacin	7246	1.6	1.5 *	2.4	952	3.9	3.3	5.2	573	0.7	0.6	0.0	100	2.0	0.0	3.3	139	4.3	2.3	7.5	104	1.9	1.4	3.1	424	17.3	13.6	19.9
		Gentamicin	-	-	-	-	-	-	-	-	-	-	-	-	-	-	-	-	-	-	-	-	-	-	-	-	425	31.1	27.9	33.3
		Tobramycin	-	-	-	-	-	-	-	-	-	-	-	-	-	-	-	-	-	-	-	-	-	-	-	-	416	21.2	15.5 *	25.2
β-lactam	Carbapenems	Imipenem	-	-	-	-	-	-	-	-	-	-	-	-	-	-	-	-	-	-	-	-	-	-	-	-	430	15.3	17.4 *	26.0
	Cephalosporins (first G)	Cefazolin	9474	14.9	13.1 *	24.7	1105	39.3	31.8 *	58.6	737	15.2	14.0	18.8	111	100.0	100.0	100.0	125	100.0	100.0	100.0	102	32.4	28.2	41.9	-	-	-	-
	Cephalosporins (second G)	Cefuroxime	10,814	10.0	8.5 *	17.9	1368	33.4	26.7 *	52.2	848	4.0	3.3	6.3	124	100.0	100.0	100.0	144	77.8	75.6	81.5	120	16.7	15.0	20.0	-	-	-	-
	Cephalosporins (third G)	Cefotaxime	10,796	7.2	6.1 *	13.2	1360	28.8	22.8 *	45.7	844	1.5	1.6	1.5	123	4.9	4.5	5.6	142	21.1	13.5 *	34.0	120	12.5	10.0	17.5	-	-	-	-
		Ceftazidime	-	-	-	-	-	-	-	-	-	-	-	-	-	-	-	-	-	-	-	-	-	-	-	-	431	26.9	24.3	28.9
	Cephalosporins (fourth G)	Cefepime	-	-	-	-	-	-	-	-	-	-	-	-	-	-	-	-	-	-	-	-	-	-	-	-	431	20.6	19.5	21.5
	Monobactams	Aztreonam	-	-	-	-	-	-	-	-	-	-	-	-	-	-	-	-	-	-	-	-	-	-	-	-	428	16.4	17.5	25.5
	Penicillins	Amoxicillin	10,816	46.3	44.1 *	57.7	1366	100.0	100.0	100.0	847	36.6	34.3 *	43.7	124	100.0	100.0	100.0	144	100.0	100.0	100.0	120	100.0	100.0	100.0	-	-	-	-
		AMX-CLA	10,807	20.3	18.8 *	28.5	1365	69.4	65.7 *	79.7	846	9.9	7.8 *	16.6	124	58.9	56.1	62.1	144	100.0	100.0	100.0	120	55.0	51.3	62.5	-	-	-	-
		PIP-TAZ	-	-	-	-	-	-	-	-	-	-	-	-	-	-	-	-	-	-	-	-	-	-	-	-	432	20.1	17.3	22.3
Quinolones		Ciprofloxacin	10,489	20.5	18.0 *	33.7	1351	34.6	27.8 *	53.7	788	21.6	18.6 *	31.0	120	42.5	42.2	42.9	142	21.1	11.4 *	37.0	119	20.2	16.3	28.2	430	44.0	39.9	47.0
Miscellaneous agents		Nitrofurantoin	10,804	7.0	6.4 *	10.2	1273	81.7	79.8 *	86.8	843	100.0	100.0	100.0	124	100.0	100.0	100.0	138	73.9	72.1	76.9	113	77.9	73.7	86.5	-	-	-	-
		Fosfomycin	9829	1.4	1.4	1.4	-	-	-	-	-	-	-	-	-	-	-	-	-	-	-	-	-	-	-	-	-	-	-	-
		SXT	10,811	24.8	23.3 *	33.1	1365	36.5	30.4 *	53.3	846	28.8	28.2	30.7	124	41.9	47.0	36.2	144	22.2	15.6 *	33.3	120	22.5	18.8	30.0	-	-	-	-

* Statistically significant differences (*p*-value < 0.05) of antimicrobial resistance between female and male patients; -, antimicrobial not tested; G, generation.

**Table 3:** Average antimicrobial resistance of the main Gram-positive uropathogens for female and male patients.

Antimicrobial Group		Antimicrobials	*E. faecalis*				*S. aureus*				*S. saprophyticus*				*S. agalactiae*			
			N	%	F	M	N	%	F	M	N	%	F	M	N	%	F	M
Aminoglycosides		Gentamicin	473	37.8	33.0	41.8	270	4.1	2.0 *	10.4	-	-	-	-	-	-	-	-
β-lactam	Cephalosporins (third G)	Cefotaxime	-	-	-	-	-	-	-	-	-	-	-	-	176	2.8	2.8	2.9
	Penicillins	Ampicillin	465	7.5	8.1	7.1	-	-	-	-	-	-	-	-	175	1.1	0.7	3.0
		Amoxicillin	436	8.3	8.2	8.3	-	-	-	-	103	48.5	47.4	66.7	166	2.4	2.3	3.0
		AMX-CLA	-	-	-	-	379	11.6	6.4 *	29.8	120	6.7	6.1	16.7	-	-	-	-
Quinolones		Ciprofloxacin	485	46.4	43.1	49.1	363	15.7	10.3 *	33.7	114	5.3	4.7	14.3	-	-	-	-
		Levofloxacin	472	38.3	29.0 *	45.8	-	-	-	-	-	-	-	-	173	7.5	7.1	9.4
Miscellaneous agents		Nitrofurantoin	470	3.2	3.3	3.1	374	5.9	2.7 *	17.7	120	4.2	3.5	14.3	146	0.7	0.9	0.0
		SXT	-	-	-	-	383	14.6	9.7 *	31.8	120	10.8	10.5	16.7	134	39.6	38.0	46.2

* Statistically significant differences (*p*-value < 0.05) of antimicrobial resistance between female and male patients; -, antimicrobial not tested. For *E. faecalis,* a higher dose of gentamicin was used. G, generation.

**Table 4:** Calculated bacterial resistance to the antimicrobials recommended as first-line therapy for empirical treatment of UTI.

		Resistance to First-Line Therapy			
Bacteria	Incidence	FOM (%)	FOM (%) ^1^	NIT (%)	NIT (%) ^1^
*E. coli*	70.1	1.4	1.0	7.0	4.9
*K. pneumoniae*	8.9	-	-	81.7	7.3
*P. mirabilis*	5.5	-	-	100.0	5.4
*E. faecalis*	3.2	-	-	3.2	0.1
*S. aureus*	2.5	-	-	5.9	0.1
*S. agalactiae*	1.1	-	-	0.7	0.0
*Enterobacter* spp.	0.9	-	-	73.9	0.7
*P. vulgaris*	0.8	-	-	100.0	0.8
*S. saprophyticus*	0.8	-	-	4.2	0.0
*K. oxytoca*	0.8	-	-	77.9	0.6
Average (%)	-	-	1.0	-	19.9

FOM, fosfomycin; NIT, nitrofurantoin; (%), average resistance; (%) ^1^, calculated resistance.

**Table 5:** Calculated bacterial resistance to the antimicrobials recommended as alternative therapy for empirical treatment of UTI.

		Resistance to Alternative Therapy											
Bacteria	Incidence	CEP 1st (%)	CEP 1st (%) ^1^	CEP 2nd (%)	CEP 2nd (%) ^1^	CEP 3rd (%)	CEP 3rd (%) ^1^	QUI (%)	QUI (%) ^1^	AMX-CLA (%)	AMX-CLA (%) ^1^	SXT (%)	SXT (%) ^1^
*E. coli*	70.1	14.9	10.4	10.0	7.0	7.2	5.0	20.5	14.4	20.3	14.2	24.8	17.4
*K. pneumoniae*	8.9	39.3	3.5	33.4	3.3	28.8	2.6	34.6	3.1	69.4	6.2	36.5	3.2
*P. mirabilis*	5.5	15.2	0.8	4.0	0.2	1.5	0.0	21.6	1.2	9.9	0.5	28.8	1.6
*E. faecalis*	3.2	-	-	-	-	-	-	42.4	1.4	-	-	-	-
*P. aeruginosa*	2.8	-	-	-	-	-	-	44.0	1.2	-	-	-	-
*S. aureus*	2.5	-	-	-	-	-	-	15.7	0.4	11.6	0.3	14.6	0.4
*S. agalactiae*	1.1	-	-	-	-	2.8	0.0	7.5	0.1	-	-	39.6	0.4
*Enterobacter* spp.	0.9	100.0	0.9	77.8	0.7	21.1	0.2	21.1	0.2	100.0	0.9	22.2	0.2
*P. vulgaris*	0.8	100.0	0.8	100.0	0.8	4.9	0.0	42.5	0.3	58.9	0.5	41.9	0.3
*S. saprophyticus*	0.8	-	-	-	-	-	-	5.3	0.0	6.7	0.1	10.8	0.1
*K. oxytoca*	0.8	32.4	0.3	16.7	0.1	12.5	0.1	20.2	0.2	55.0	0.4	22.5	0.2
Average (%)	-	-	16.7	-	12.1	-	7.9	-	22.5	-	23.1	-	23.8

CEP, cephalosporin; QUI, quinolones; AMX-CLA, amoxicillin and clavulanic acid; SXT, trimethoprim-sulfamethoxazole; (%), average resistance; (%) ^1^, calculated resistance.

**Table 6:** Percentage of multidrug resistance in the studied bacteria.

Bacteria in UTI	% of MDR Isolates
*E. coli*	23.3
*K. pneumoniae*	40.4
*P. mirabilis*	10.0
*P. vulgaris*	19.4
*Enterobacter* spp.	29.9
*K. oxytoca*	24.2
*P. aeruginosa*	34.7
*S. aureus*	18.3
*S. saprophyticus*	5.8
*S. agalactiae*	6.8

## Data Availability

Not applicable.
